# Supplementary material for: Quantifying institutional-level length of stay variation among hospitalizations for schizophrenia in Ontario between 2014–2021
Source: PLOS Ment Health. 2025 Aug 4;2(8):e0000391. doi: 10.1371/journal.pmen.0000391 (PMC12798577; doi:10.1371/journal.pmen.0000391)
Supplement: S1 Appendix — (DOCX) [file pmen.0000391.s001.docx]

# S1 Appendix. Additional Analyses and Model Coefficients.

Table of Contents

[S1 Appendix. Additional Analyses and Model Coefficients. 1](#_Toc194395050)

[Stratification Analyses 2](#_Toc194395051)

[Table A. Stratification Analysis Result Comparisons in Large Community Hospitals 2](#_Toc194395052)

[Table B. Stratification Analysis Result Comparisons in Specialty Mental Health Hospitals. 3](#_Toc194395053)

[Table C. Stratification Analysis Result Comparisons in Teaching Hospitals. 4](#_Toc194395054)

[Sensitivity Analyses 5](#_Toc194395055)

[Table D. Sensitivity Analysis Result Comparisons in Large Community Hospitals. 5](#_Toc194395056)

[Table E. Sensitivity Analysis Result Comparisons in Specialty MH Hospitals. 6](#_Toc194395057)

[Table F. Sensitivity Analysis Result Comparisons in Teaching Hospitals. 7](#_Toc194395058)

[Coefficients of Mixed Effects Models 8](#_Toc194395059)

[Table G. Coefficients of Mixed Effects Model of log(Total LOS) in Large Community Hospitals. 8](#_Toc194395060)

[Table H. Coefficients of Mixed Effects Model of log(Total LOS) in Specialty MH Hospitals. 10](#_Toc194395061)

[Table I. Coefficients of Mixed Effects Model of log(Total LOS) in Teaching Hospitals. 12](#_Toc194395062)

## Stratification Analyses

### Table A. Stratification Analysis Result Comparisons in Large Community Hospitals

|  | **Overall** | **Age Stratified** | | **Sex Stratified** | | **Obs. per Unique Individual Stratified** | |
| --- | --- | --- | --- | --- | --- | --- | --- |
|  | Total LOS (Primary) | Total LOS: >=50 | Total LOS: <50 | Total LOS: Females | Total LOS: Males | Total LOS: 1 obs. | Total LOS: >1 obs. |
| Number of Hospitals (clusters) | 59 | 59 | 59 | 59 | 59 | 58 | 59 |
| Hospital-level Variation | 0.248 | 0.248 | 0.283 | 0.219 | 0.238 | 0.250 | 0.325 |
| Individual-level Variation | 0.599 | 0.599 | 0.681 | 0.562 | 0.625 | 0.579 | 0.588 |
| Total Variation | 0.847 | 0.847 | 0.964 | 0.781 | 0.863 | 0.830 | 0.913 |
| % Hospital-Level Variation | 29.30 | 29.30 | 29.34 | 28.04 | 27.58 | 30.19 | 35.62 |
| pseudo r2 (Fixed Effects) | 0.08 | 0.08 | 0.07 | 0.07 | 0.08 | 0.07 | 0.08 |
| pseudo r2 (Fixed + Random Effects) | 0.35 | 0.35 | 0.34 | 0.33 | 0.33 | 0.35 | 0.41 |
| Residual SE | 30.15 | 30.15 | 16.95 | 24.80 | 20.05 | 23.48 | 16.62 |

### Table B. Stratification Analysis Result Comparisons in Specialty Mental Health Hospitals.

|  | **Overall** | **Age Stratified** | | **Sex Stratified** | | **Obs. per Unique Individual Stratified** | |
| --- | --- | --- | --- | --- | --- | --- | --- |
|  | Total LOS (Primary) | Total LOS: >=50 | Total LOS: <50 | Total LOS: Females | Total LOS: Males | Total LOS: 1 obs. | Total LOS: >1 obs. |
| Number of Hospitals (clusters) | *s* | *s* | *s* | *s* | *s* | *s* | *s* |
| Hospital-level Variation | 0.276 | 0.149 | 0.375 | 0.292 | 0.267 | 0.292 | 0.271 |
| Individual-level Variation | 1.152 | 1.231 | 1.096 | 1.122 | 1.164 | 1.096 | 1.272 |
| Total Variation | 1.429 | 1.380 | 1.471 | 1.414 | 1.431 | 1.388 | 1.543 |
| % Hospital-Level Variation | 19.34 | 10.81 | 25.50 | 20.67 | 18.66 | 21.06 | 17.54 |
| pseudo r2 (Fixed Effects) | 0.24 | 0.29 | 0.21 | 0.26 | 0.24 | 0.24 | 0.26 |
| pseudo r2 (Fixed + Random Effects) | 0.39 | 0.36 | 0.41 | 0.41 | 0.38 | 0.40 | 0.39 |
| Residual SE | 17.57 | 10.37 | 14.11 | 10.90 | 14.27 | 14.64 | 9.52 |

*s =* supressed due to cell size < 6

### Table C. Stratification Analysis Result Comparisons in Teaching Hospitals.

|  | **Overall** | **Age Stratified** | | **Sex Stratified** | | **Obs. per Unique Individual Stratified** | |
| --- | --- | --- | --- | --- | --- | --- | --- |
|  | Total LOS (Primary) | Total LOS: >=50 | Total LOS: <50 | Total LOS: Females | Total LOS: Males | Total LOS: 1 obs. | Total LOS: >1 obs. |
| Number of Hospitals (clusters) | 17 | 17 | 17 | 17 | 17 | 17 | 17 |
| Hospital-level Variation | 0.195 | 0.246 | 0.188 | 0.213 | 0.212 | 0.172 | 0.318 |
| Individual-level Variation | 0.794 | 0.860 | 0.755 | 0.801 | 0.787 | 0.796 | 0.775 |
| Total Variation | 0.989 | 1.106 | 0.943 | 1.014 | 0.999 | 0.968 | 1.093 |
| % Hospital-Level Variation | 19.73 | 22.25 | 19.90 | 21.00 | 21.20 | 17.79 | 29.09 |
| pseudo r2 (Fixed Effects) | 0.15 | 0.12 | 0.15 | 0.13 | 0.15 | 0.15 | 0.14 |
| pseudo r2 (Fixed + Random Effects) | 0.32 | 0.31 | 0.32 | 0.32 | 0.33 | 0.30 | 0.39 |
| Residual SE | 18.18 | 10.72 | 14.57 | 11.92 | 14.28 | 14.73 | 10.50 |

## Sensitivity Analyses

### Table D. Sensitivity Analysis Result Comparisons in Large Community Hospitals.

|  | **Model Variant** | | | | |
| --- | --- | --- | --- | --- | --- |
|  | **Total LOS (Primary)** | **Total LOS: Fewer Fixed Effects** | **Acute LOS** | **Total LOS: Tukey Bounded** | **Total LOS: No ALC Days** |
| Number of Hospitals (clusters) | 59 | 59 | 59 | 59 | 59 |
| Hospital-level Variation | 0.248 | 0.262 | 0.236 | 0.041 | 0.214 |
| Individual-level Variation | 0.599 | 0.655 | 0.590 | 0.429 | 0.576 |
| Total Variation | 0.847 | 0.918 | 0.826 | 0.470 | 0.790 |
| % Hospital-Level Variation | 29.30 | 28.58 | 28.53 | 8.70 | 27.08 |
| pseudo r2 (Fixed Effects) | 0.08 | 0.01 | 0.08 | 0.11 | 0.08 |
| pseudo r2 (Fixed + Random Effects) | 0.35 | 0.29 | 0.34 | 0.19 | 0.33 |
| Residual SE | 30.15 | 57.60 | 29.93 | 24.48 | 29.39 |

### Table E. Sensitivity Analysis Result Comparisons in Specialty MH Hospitals.

|  | **Model Variant** | | | | |
| --- | --- | --- | --- | --- | --- |
|  | **Total LOS (Primary)** | **Total LOS: Less Fixed Effects** | **Acute LOS** | **Total LOS: Tukey Bounded** | **Total LOS: No ALC Days** |
| Number of Hospitals (clusters) | *s* | *s* | *s* | *s* | *s* |
| Hospital-level Variation | 0.276 | 0.447 | 0.268 | 0.202 | 0.289 |
| Individual-level Variation | 1.152 | 1.552 | 1.105 | 0.770 | 0.962 |
| Total Variation | 1.429 | 1.999 | 1.373 | 0.972 | 1.251 |
| % Hospital-Level Variation | 19.34 | 22.34 | 19.51 | 20.74 | 23.11 |
| pseudo r2 (Fixed Effects) | 0.24 | 0.01 | 0.08 | 0.29 | 0.25 |
| pseudo r2 (Fixed + Random Effects) | 0.39 | 0.23 | 0.34 | 0.44 | 0.42 |
| Residual SE | 17.57 | 37.29 | 29.93 | 13.61 | 15.57 |

*s =* supressed due to cell size < 6

### Table F. Sensitivity Analysis Result Comparisons in Teaching Hospitals.

|  | **Model Variant** | | | | |
| --- | --- | --- | --- | --- | --- |
|  | **Total LOS (Primary)** | **Total LOS: Fewer Fixed Effects** | **Acute LOS** | **Total LOS: Tukey Bounded** | **Total LOS: No ALC Days** |
| Number of Hospitals (clusters) | 17 | 17 | 17 | 17 | 17 |
| Hospital-level Variation | 0.195 | 0.218 | 0.192 | 0.095 | 0.179 |
| Individual-level Variation | 0.794 | 0.933 | 0.774 | 0.614 | 0.747 |
| Total Variation | 0.989 | 1.150 | 0.966 | 0.709 | 0.926 |
| % Hospital-Level Variation | 19.73 | 18.93 | 19.87 | 13.41 | 19.28 |
| pseudo r2 (Fixed Effects) | 0.15 | 0.01 | 0.15 | 0.18 | 0.15 |
| pseudo r2 (Fixed + Random Effects) | 0.32 | 0.20 | 0.32 | 0.29 | 0.32 |
| Residual SE | 18.18 | 36.00 | 17.95 | 15.28 | 17.40 |

## Coefficients of Mixed Effects Models

### Table G. Coefficients of Mixed Effects Model of log(Total LOS) in Large Community Hospitals.

| **Type of Effect** | **Group** | **Term** | **Estimate** | **SE** | **Statistic** |
| --- | --- | --- | --- | --- | --- |
| Fixed | - | (Intercept) | 2.223 | 0.082 | 27.10 |
| Fixed | - | Age at Admit | 0.007 | 0.000 | 26.63 |
| Fixed | - | Admin. Sex: Intersex | -0.475 | 0.274 | -1.73 |
| Fixed | - | Admin. Sex: Male | -0.056 | 0.007 | -8.08 |
| Fixed | - | SAC Level: CA | 0.003 | 0.017 | 0.16 |
| Fixed | - | SAC Level: Outside CMA | 0.014 | 0.016 | 0.84 |
| Fixed | - | SAC Level: Unknown/Missing | -0.010 | 0.040 | -0.26 |
| Fixed | - | Neighbourhood Income Quintile: 2nd | 0.010 | 0.009 | 1.07 |
| Fixed | - | Neighbourhood Income Quintile: 3rd | 0.026 | 0.010 | 2.69 |
| Fixed | - | Neighbourhood Income Quintile: 4th | 0.030 | 0.010 | 2.87 |
| Fixed | - | Neighbourhood Income Quintile: 5th - Highest Income Quintile | 0.044 | 0.012 | 3.77 |
| Fixed | - | Neighbourhood Income Quintile: Unknown/Missing | 0.031 | 0.012 | 2.51 |
| Fixed | - | PSS-Long at Admit | 0.013 | 0.001 | 14.99 |
| Fixed | - | Education Level: 8th Grade or Less | 0.058 | 0.047 | 1.22 |
| Fixed | - | Education Level: 9th to 11th Grade | 0.023 | 0.045 | 0.51 |
| Fixed | - | Education Level: Completed High School | 0.029 | 0.045 | 0.64 |
| Fixed | - | Education Level: Some College or University | 0.029 | 0.045 | 0.65 |
| Fixed | - | Education Level: Technical or Trade School | -0.017 | 0.049 | -0.35 |
| Fixed | - | Education Level: Diploma or Bachelor's Degree | 0.021 | 0.046 | 0.47 |
| Fixed | - | Education Level: Graduate Degree or Higher | 0.008 | 0.051 | 0.15 |
| Fixed | - | Education Level: Unknown/Missing | 0.028 | 0.045 | 0.62 |
| Fixed | - | Income Source: Insurance/Social Assistance | 0.030 | 0.008 | 3.60 |
| Fixed | - | Income Source: Other | 0.063 | 0.012 | 5.20 |
| Fixed | - | Income Source: No Income | 0.111 | 0.011 | 9.92 |
| Fixed | - | Income Source: Unknown/Missing | -0.333 | 0.048 | -6.96 |
| Fixed | - | Recent Psychiatric Admission: 1 or 2 Admits in 2 Years | 0.079 | 0.008 | 9.99 |
| Fixed | - | Recent Psychiatric Admission: > 2 Admits in 2 Years | 0.093 | 0.009 | 10.20 |
| Fixed | - | Recent Psychiatric Admission: Unknown/Missing | -0.149 | 0.068 | -2.21 |
| Fixed | - | Recent Community MH Contact: > 30 Days | 0.027 | 0.008 | 3.14 |
| Fixed | - | Recent Community MH Contact: None | -0.004 | 0.008 | -0.55 |
| Fixed | - | Recent Community MH Contact: Unknown/Missing | -1.187 | 0.069 | -17.14 |
| Fixed | - | Addictions-Related Admission: No | 0.087 | 0.009 | 10.07 |
| Fixed | - | Marital Status: Divorced | 0.049 | 0.015 | 3.23 |
| Fixed | - | Marital Status: Partner/Significant Other | -0.019 | 0.022 | -0.85 |
| Fixed | - | Marital Status: Separated | 0.057 | 0.019 | 3.03 |
| Fixed | - | Marital Status: Single (Never Married) | 0.096 | 0.011 | 9.09 |
| Fixed | - | Marital Status: Widowed | 0.175 | 0.024 | 7.31 |
| Fixed | - | Marital Status: Unknown/Missing | -0.045 | 0.061 | -0.74 |
| Fixed | - | Temporary Address at Admission: No | -0.002 | 0.008 | -0.26 |
| Fixed | - | Temporary Address at Admission: Unknown/Missing | -0.368 | 0.045 | -8.15 |
| Random | Hospital | Standard Dev. (Intercept) | 0.498 | N/A | N/A |
| Random | Residual | Standard Dev. (Observation) | 0.774 | N/A | N/A |

### Table H. Coefficients of Mixed Effects Model of log(Total LOS) in Specialty MH Hospitals.

| **Type of Effect** | **Group** | **Term** | **Estimate** | **SE** | **Statistic** |
| --- | --- | --- | --- | --- | --- |
| Fixed | - | (Intercept) | 3.193 | 0.328 | 9.74 |
| Fixed | - | Age at Admit | 0.011 | 0.001 | 12.95 |
| Fixed | - | Admin. Sex: Intersex | 0.247 | 0.381 | 0.65 |
| Fixed | - | Admin. Sex: Male | -0.022 | 0.023 | -0.97 |
| Fixed | - | SAC Level: CA | -0.162 | 0.051 | -3.18 |
| Fixed | - | SAC Level: Outside CMA | -0.084 | 0.053 | -1.57 |
| Fixed | - | SAC Level: Unknown/Missing | -0.054 | 0.096 | -0.56 |
| Fixed | - | Neighbourhood Income Quintile: 2nd | 0.035 | 0.031 | 1.13 |
| Fixed | - | Neighbourhood Income Quintile: 3rd | 0.012 | 0.034 | 0.36 |
| Fixed | - | Neighbourhood Income Quintile: 4th | 0.033 | 0.034 | 0.96 |
| Fixed | - | Neighbourhood Income Quintile: 5th - Highest Income Quintile | 0.039 | 0.035 | 1.14 |
| Fixed | - | Neighbourhood Income Quintile: Unknown/Missing | -0.035 | 0.036 | -0.98 |
| Fixed | - | PSS-Long at Admit | -0.002 | 0.003 | -0.65 |
| Fixed | - | Education Level: 8th Grade or Less | -0.247 | 0.223 | -1.11 |
| Fixed | - | Education Level: 9th to 11th Grade | -0.176 | 0.217 | -0.81 |
| Fixed | - | Education Level: Completed High School | -0.280 | 0.216 | -1.30 |
| Fixed | - | Education Level: Some College or University | -0.326 | 0.216 | -1.51 |
| Fixed | - | Education Level: Technical or Trade School | -0.185 | 0.228 | -0.81 |
| Fixed | - | Education Level: Diploma or Bachelor's Degree | -0.324 | 0.222 | -1.46 |
| Fixed | - | Education Level: Graduate Degree or Higher | -0.422 | 0.220 | -1.92 |
| Fixed | - | Education Level: Unknown/Missing | -0.360 | 0.217 | -1.66 |
| Fixed | - | Income Source: Insurance/Social Assistance | 0.178 | 0.034 | 5.19 |
| Fixed | - | Income Source: Other | 0.012 | 0.044 | 0.28 |
| Fixed | - | Income Source: No Income | 0.039 | 0.035 | 1.09 |
| Fixed | - | Income Source: Unknown/Missing | 0.565 | 0.348 | 1.62 |
| Fixed | - | Recent Psychiatric Admission: 1 or 2 Admits in 2 Years | 0.210 | 0.027 | 7.85 |
| Fixed | - | Recent Psychiatric Admission: > 2 Admits in 2 Years | 0.319 | 0.032 | 10.03 |
| Fixed | - | Recent Psychiatric Admission: Unknown/Missing | -0.290 | 0.421 | -0.69 |
| Fixed | - | Recent Community MH Contact: > 30 Days | 0.059 | 0.028 | 2.10 |
| Fixed | - | Recent Community MH Contact: None | -0.029 | 0.030 | -0.99 |
| Fixed | - | Recent Community MH Contact: Unknown/Missing | -0.463 | 0.375 | -1.23 |
| Fixed | - | Addictions-Related Admission: No | 0.178 | 0.026 | 6.96 |
| Fixed | - | Marital Status: Divorced | 0.121 | 0.057 | 2.14 |
| Fixed | - | Marital Status: Partner/Significant Other | -0.167 | 0.094 | -1.78 |
| Fixed | - | Marital Status: Separated | 0.145 | 0.073 | 1.98 |
| Fixed | - | Marital Status: Single (Never Married) | 0.184 | 0.043 | 4.29 |
| Fixed | - | Marital Status: Widowed | 0.079 | 0.094 | 0.84 |
| Fixed | - | Marital Status: Unknown/Missing | -0.042 | 0.351 | -0.12 |
| Fixed | - | Temporary Address at Admission: No | -0.075 | 0.030 | -2.51 |
| Fixed | - | Temporary Address at Admission: Unknown/Missing | -2.088 | 0.179 | -11.65 |
| Random | Hospital | Standard Dev. (Intercept) | 0.526 | N/A | N/A |
| Random | Residual | Standard Dev. (Observation) | 1.074 | N/A | N/A |

### Table I. Coefficients of Mixed Effects Model of log(Total LOS) in Teaching Hospitals.

| **Type of Effect** | **Group** | **Term** | **Estimate** | **SE** | **Statistic** |
| --- | --- | --- | --- | --- | --- |
| Fixed | - | (Intercept) | 2.361 | 0.120 | 19.76 |
| Fixed | - | Age at Admit | 0.007 | 0.001 | 13.49 |
| Fixed | - | Admin. Sex: Intersex | -0.366 | 0.338 | -1.08 |
| Fixed | - | Admin. Sex: Male | -0.067 | 0.015 | -4.54 |
| Fixed | - | SAC Level: CA | 0.138 | 0.057 | 2.42 |
| Fixed | - | SAC Level: Outside CMA | -0.016 | 0.037 | -0.42 |
| Fixed | - | SAC Level: Unknown/Missing | -0.040 | 0.065 | -0.63 |
| Fixed | - | Neighbourhood Income Quintile: 2nd | 0.001 | 0.020 | 0.05 |
| Fixed | - | Neighbourhood Income Quintile: 3rd | -0.002 | 0.022 | -0.08 |
| Fixed | - | Neighbourhood Income Quintile: 4th | 0.003 | 0.024 | 0.14 |
| Fixed | - | Neighbourhood Income Quintile: 5th - Highest Income Quintile | 0.085 | 0.026 | 3.34 |
| Fixed | - | Neighbourhood Income Quintile: Unknown/Missing | 0.023 | 0.024 | 0.95 |
| Fixed | - | PSS-Long at Admit | 0.015 | 0.002 | 7.90 |
| Fixed | - | Education Level: 8th Grade or Less | 0.102 | 0.049 | 2.08 |
| Fixed | - | Education Level: 9th to 11th Grade | 0.086 | 0.032 | 2.70 |
| Fixed | - | Education Level: Completed High School | 0.077 | 0.028 | 2.77 |
| Fixed | - | Education Level: Some College or University | 0.102 | 0.031 | 3.26 |
| Fixed | - | Education Level: Technical or Trade School | -0.053 | 0.051 | -1.05 |
| Fixed | - | Education Level: Diploma or Bachelor's Degree | 0.042 | 0.036 | 1.19 |
| Fixed | - | Education Level: Graduate Degree or Higher | 0.148 | 0.060 | 2.45 |
| Fixed | - | Education Level: Unknown/Missing | 0.070 | 0.029 | 2.42 |
| Fixed | - | Income Source: Insurance/Social Assistance | -0.030 | 0.019 | -1.62 |
| Fixed | - | Income Source: Other | 0.009 | 0.029 | 0.31 |
| Fixed | - | Income Source: No Income | 0.101 | 0.026 | 3.87 |
| Fixed | - | Income Source: Unknown/Missing | -1.018 | 0.092 | -11.04 |
| Fixed | - | Recent Psychiatric Admission: 1 or 2 Admits in 2 Years | 0.059 | 0.017 | 3.50 |
| Fixed | - | Recent Psychiatric Admission: > 2 Admits in 2 Years | 0.026 | 0.021 | 1.25 |
| Fixed | - | Recent Psychiatric Admission: Unknown/Missing | 0.456 | 0.222 | 2.06 |
| Fixed | - | Recent Community MH Contact: > 30 Days | 0.044 | 0.019 | 2.33 |
| Fixed | - | Recent Community MH Contact: None | 0.006 | 0.018 | 0.32 |
| Fixed | - | Recent Community MH Contact: Unknown/Missing | -1.073 | 0.226 | -4.75 |
| Fixed | - | Addictions-Related Admission: No | 0.146 | 0.019 | 7.79 |
| Fixed | - | Marital Status: Divorced | 0.178 | 0.036 | 4.98 |
| Fixed | - | Marital Status: Partner/Significant Other | -0.008 | 0.058 | -0.14 |
| Fixed | - | Marital Status: Separated | 0.031 | 0.046 | 0.67 |
| Fixed | - | Marital Status: Single (Never Married) | 0.157 | 0.025 | 6.32 |
| Fixed | - | Marital Status: Widowed | 0.117 | 0.058 | 2.01 |
| Fixed | - | Marital Status: Unknown/Missing | 0.209 | 0.351 | 0.59 |
| Fixed | - | Temporary Address at Admission: No | -0.017 | 0.017 | -1.00 |
| Fixed | - | Temporary Address at Admission: Unknown/Missing | -0.538 | 0.100 | -5.40 |
| Random | Hospital | Standard Dev. (Intercept) | 0.442 | N/A | N/A |
| Random | Residual | Standard Dev. (Observation) | 0.891 | N/A | N/A |
