# Supplementary material for: Quantifying institutional-level length of stay variation among hospitalizations for schizophrenia in Ontario between 2014–2021
Source: PLOS Ment Health. 2025 Aug 4;2(8):e0000391. doi: 10.1371/journal.pmen.0000391 (PMC12798577; doi:10.1371/journal.pmen.0000391)
Supplement: Attachment — Submitted filename: PLOS-MH Reviewer Responses-Apr10.docx [file pmen.0000391.s004.docx]

We thank the reviewer for their very thoughtful comments on our manuscript. In particular, we are grateful for the reviewer’s attention to detail, which uncovered important discrepancies in the reporting of our findings. We have also added a sensitivity analysis to test the reviewer’s concern about repeat hospitalizations. Their comments also helped us improve our discussion of the study’s implications for the mental health sector in Ontario. We believe these changes have significantly improved our manuscript.

***Reviewer Comment:*** *For the audience’s benefit, it would be useful to list the 5 specialty mental health hospitals captured in the study, the city they are found in, and any unique differences that distinguish their intake process from academic and community hospitals (in general). A 2016 report by the Office of the Auditor General of Ontario lists 4 specialty hospitals (CAMH - Toronto, Ontario Shores - Whitby, The Royal - Ottawa, and Waypoint - Penetanguishene), while an online search also adds St. Joseph’s – London and North Bay Regional (neither making a list of 5):* [*https://www.auditor.on.ca/en/content/annualreports/arreports/en16/v1_312en16.pdf*](https://www.auditor.on.ca/en/content/annualreports/arreports/en16/v1_312en16.pdf) *. It is possible that some began operating later in the observation period, and this information as well as other contextual details are important to readers.\*

**Author Response:** The specialty mental health hospitals in our data are all regulated under psychiatric inpatient facilities according to Ontario’s Mental Health Act. This list is based on the facility classification of "SPECIALTY MH HOSP" in the Discharge Abstract Database and the Ontario Mental Health Reporting System, which have schizophrenia inpatient records during our study period and meet our exclusion criteria.

Unfortunately, the data steward—the Ontario Ministry of Health—strictly prohibits the identification of specific facilities or the reporting of cell sizes smaller than six. Thus, we are not permitted to identify the specific hospitals included in the data. This was an oversight in our initial submission, and any mention of there being five specialty hospitals has been removed from the manuscript, and the corresponding cells have been suppressed in the tables.

However, we do want to address the reviewer’s concern about the different intake processes that various hospitals may have for patients with a diagnosis of schizophrenia. We have done this in our response to similar issues raised by the reviewer in their final comment (see below).

***Reviewer Comment:*** *The authors report that they include two types of primary diagnosis of schizophrenia. The first (and obvious) is where inpatients’ primary diagnosis is defined as schizophrenia as intake (i.e., time at admission); while the second is prior to discharge. One thing I would have liked to see, as part of stratified analysis (and appreciate the inclusion of other such analyses), is models run using this distinction (i.e., comparing hospitalizations where at time of admission schizophrenia was the primary cause compared with at time of discharge). Given the authors retained the longest stay as the outcome for their analysis, it is plausible that state at intake and transfer policies may explain differences in the kinds of patients hospitals treat and were not controlled for with fixed effects…*

**Author Response:** Five hundred and sixty-seven (567) hospitalizations (4.3% of hospitalization episodes that involved a transfer; 0.6% of all 89,186 hospitalization episodes in the analysis) did not have a primary diagnosis of schizophrenia during their initial length of stay. Thus unfortunately, there are not enough observations to conduct a stratified sensitivity analysis for this subgroup, however, the low prevalence of this suggests that it is unlikely to have had an effect on our analysis.

For further context, of those 567 hospitalization episodes where a patient experienced a transfer and did not receive a diagnosis of schizophrenia during their initial length of stay, 224 of those hospitalization episodes had a schizophrenia diagnosis attributed to their longest length of stay portion within that episode (the portion used in our analysis) (n = 224, 39.5% of the 567 episodes, 0.2% of all episodes). In the remaining cases, the longest length of stay portion had primary diagnoses of bipolar disorder (n= 72, 12.7% of the 567 episodes, 0.1% of all episodes), substance abuse (n= 83, 14.6% of the 567 episodes, 0.1% of all episodes), mood disorders (n =92, 16.2% of the 567 episodes, 0.1% of all episodes), or other diagnoses (n=96, 16.9% of the 567 episodes, 0.1% of all episodes).

***Reviewer Comment:*** *Generally speaking, the authors do a nice job of defining every term they include in their models. I may have missed it, but I would appreciate the inclusion of definitions for ‘recent psychiatric admission’, ‘recent community mental health contact’, and ‘additions-related admission’. Specifically, aside from the information included in appendix 1 (e.g., recent psychiatric admission: 1 or 2 admits in 2 years) I’d like to know what data were used to create these flags, and in the case of ‘addictions-related admission’ clarification on if this measure was flagging additional codes used for the episode of schizophrenia or prior to the event, and if prior – how long in the past. Similarly for the other two definitions, how long a look back was used and ICD codes considered.*

**Author Response:** These variables are all from the Resident Assessment Instrument - Mental Health (RAI-MH) and are self-reported by the patient as part of completing the RAI-MH. We have updated the following lines 161-163 to more clearly reflect this: [changes in square brackets]

“The [self-report RAI-MH items] of recent psychiatric admission, recent community MH contact, temporary address at admission, and addiction admission were also extracted from OMHRS [as patient-level variables.[15,16]]”

***Reviewer Comment:*** *Early in the results, the authors explain how they identified 100,571 discharge records in the OMHRS, and after removing the shorter stays where a transfer occurred, and a series of cases excluded owing to invalid or missing information, they retained 89,186. I cannot make the numbers work out to this sum when 15,903 were removed as part of the process of retaining a single record per hospitalization episode. This, I feel is the biggest crux of the overall discrepancy issue I observed. While columns in tables added up upon spot checks, the number of hospitalizations retained across the different facility types did not appear to be accurate based on the description provided and the organization of the flow chart.*

**Author Response:** Thank you for catching this miscommunication and typo. The initial number of discharges identified in the Ontario Mental Health Reporting System (OHMRS) was 110,571 (not 100,571). While 25,863 *potential transfer record*s were flagged, only 20,748 met the transfer definition laid out in the paper. 11,721 of those OMHRS discharge records were removed to create the final sample of 98,850. The erroneously reported 15,903 records excluded came from subtracting the number of unique electronic health card numbers (eHNs)- rather than unique hospitalizations (the focus of interest for this study) - from the 25,863 potential transfer records. We have updated figure 1 to reflect this and have updated the manuscript lines 227 through 233 to read: [changes are in square brackets]

“From the beginning of the 2014 fiscal year through the end of the 2021 fiscal year there were [110,571] discharges [with] a primary diagnosis of schizophrenia in Ontario. Of those discharge records, we identified [20,748] records which involved at least 1 interhospital transfer [within] a single hospitalization [episode]. After selecting the record pertaining to the longest LOS portion per hospitalization [episode which had been] identified as [including at least one] transfer, [11,721] of those [20,748] transfer-related records were [removed. This resulted in a total of 98,850 unique hospitalizations being identified prior to applying the study’s exclusion criteria.]”

***Reviewer Comment:*** *Relatedly, although not mentioned in the model, it is not clear if there were patients with repeat hospitalizations, and if so – were patient repeated measures incorporated in the model (which should be the case if there was a sufficient proportion of hospitalization records that were repeat patients).*

**Author Response:** Yes, we included repeat visits as our analysis was at the level of the episode of care. It is captured by the Resident Assessment Instrument - Mental Health (RAI-MH) item of ‘Recent Psychiatric Admits`, which was included as part of the individual-level portion of the mixed effects models.

The RAI-MH also captures ‘Any Psychiatric Admits’ using the same response levels of 0, 1, 2+, and unknown/missing as the ‘Recent Psychiatric Admits’ variable. We excluded the ‘Any Psychiatric Admits’ variable from our final models due to its high correlation (r > 0.7) with the ‘Recent Psychiatric Admits’. Strong correlation between fixed effects tends to bias mixed effects model estimates, which is mentioned in lines 200-202 of the manuscript.

Forty percent (18,199 of 46,008) of unique eHNs have multiple observations in our dataset. Thus, we thought it appropriate to add a sensitivity analysis to our study where we checked whether variation in length of stay was different between those with repeat hospitalizations and those without during the study period. We added the following text to the methods (lines 210-214):

“Since some patients had repeat hospitalizations during the study period, we also conducted a sensitivity analysis in which we stratified the cohort into patients with unique hospitalizations and those with repeat hospitalizations during the study period to determine if the variation in LOS differed for these groups.”

We added the following to the results (lines 355-361):

“Finally, when we limited the analysis to patients with a single hospitalization, the variation attributable to the hospital level increased to 35% for large community hospitals, increased to 30% for teaching hospitals, and remained the same for specialized mental health hospitals. When we limited the analysis to patients with multiple hospitalizations, the variation attributable to the hospital level decreased to 26% for large community hospitals, decreased to 17% for teaching hospitals, and remained the same for specialty mental health hospitals”

The results of the added sensitivity analysis can be found in the updated Appendix S1 in Table A.

***Reviewer Comment:*** *I understand that given the descriptive nature of the paper, the authors cannot provide explanations for the variations in length of stay observed across facilities. However, given that each mental health hospital (where the variation at the hospital level was largest) is in a unique urban setting, and limited information on how intake and discharge at these sites operate – it is possible that these facilities are servicing very different patients whose differences (as briefly mentioned in the limitations) are not sufficiently accounted for. This is not to discount the authors work, but rather to support the idea that additional discussion on how these 5 sites operate are needed to then further support the idea that ‘more research is needed’ – and specifically how that additional inquiry could be structured.*

**Author Response:** Thank you for this very helpful comment. We agree that our discussion would benefit from additional nuance around the different implications our findings have for hospitals of a different type. We have added the following to the discussion section of the paper (lines 411 - 419):

“Our study highlights that the variation in LOS differs by hospital type. However, the causes of this variation and the corresponding policy solutions may differ within each hospital type. Hospitalizations for schizophrenia in large community hospitals may vary due to the heterogeneity of resources, community partnerships, staff composition, and clinical experience and expertise with severe mental illness. In contrast, specialty mental health hospitals may also be heterogeneous but differ in their institutional policies, such as how staff physicians are financially incentivized or whether they discharge patients before securing stable housing. More research is needed to determine the impact of hospital-level factors on the LOS for individuals with schizophrenia, and how these factors may vary by hospital type.”
