# Supplementary material for: Quantifying institutional-level length of stay variation among hospitalizations for schizophrenia in Ontario between 2014–2021
Source: PLOS Ment Health. 2025 Aug 4;2(8):e0000391. doi: 10.1371/journal.pmen.0000391 (PMC12798577; doi:10.1371/journal.pmen.0000391)
Supplement: Attachment — Submitted filename: PLOS-MH Reviewer Responses - Round 2.docx [file pmen.0000391.s005.docx]

We thank the reviewers for their comments on our paper. Once again, we believe they have strengthened the quality of our work. We have attempted to address these comments directly in the responses below and/or in the manuscript itself.

# Reviewer #2

## Methodology

1. Clarify Hospital Classification:

***Reviewer Comment:*** *The definition of - speciality mental health hospitals should be explicitly stated (e.g., whether they include forensic units or specific types of care).*

**Author Response:** On lines 139-141 of the manuscript, we define specialty mental health hospitals as “speciality mental health hospitals (stand-alone quaternary care hospitals that provide specialized mental health and addiction care”. In our data, all specialty mental health hospitals are stand-alone facilities regulated under psychiatric inpatient facilities according to Ontario’s Mental Health Act.

We have also updated the following lines 137-138 to help clarify the classification of hospitals in our study: [changes in square brackets]

“We captured the type of hospital using the [facility] classification [of the hospital in the

IntelliHealth data]: large community hospitals (general hospitals with…”

2. Missing Data Handling:

***Reviewer Comment:*** *Justify the assumption that missing data (e.g., education level, income quintile) are not missing at random. Consider sensitivity analyses (e.g., multiple imputation) to test robustness.*

**Author Response:**
Adding sensitivity analyses in addition to those we have already conducted (e.g. using multiple imputation) were not considered for this analysis. No data was dropped due to missing data in these missing variables, instead, we created a missing category for these variables. The individual coefficients of those variables with missing data (none of which were primary variables in our analysis) are not interpreted as part of our analysis of institutional-level variation in LOS.

In regards to our not assuming that missing data in those variables is missing at random, in table 1 we can see that the percentage of “Unknown/Missing” responses shows considerable differences between hospital types (e.g., a person’s income source is missing in 0.7% of stays in large community hospitals and is missing in 6.1% of stays in specialty mental health hospitals). This suggests that non-random factors such as hospital reporting policies or clinical environment are affecting which data is missing for person’s hospitalized for schizophrenia. We have updated the following lines 174-175 to more clearly reflect this: [changes in square brackets]

“...were not assumed to be missing at random [due to differences in missingness by hospital type], and thus were coded as a discrete `Unknown/Missing` category…”

3. Transfer Episodes:

***Reviewer Comment:*** *Provide more details on how inter-hospital transfers were identified and handled (e.g., criteria for defining a transfer and impact on LOS calculations).*

**Author Response:** Those details are provided in lines 99-103, which state:

“...in instances where an inter-hospital transfer occurred only the record with the longest LOS from that episode of hospitalization was kept, in the case of ties the most recent record was selected. An inter-hospital transfer within an episode of hospitalization was defined as when a person had been discharged from hospital and then admitted to a different hospital with a primary diagnosis of schizophrenia within 48 hours.”

## Quality of Analysis

1. Outlier Analysis:

***Reviewer Comment:*** *The outlier removal sensitivity analysis is functional, but the Tukey fence method's rationale should be explained. Consider alternative methods (e.g., truncation, winsorization) and discuss their impact.*

**Author Response:** The Tukey fence method for outlier detection is a well-established ‘rule-of-thumb’ non-parametric method of univariate outlier detection and can be represented visually using box and whisker plots. Given the magnitude of difference between outlier LOS values and the median LOS values we do not believe that using alternative methods of outlier identification would change our interpretation (lines 335-337) that “...a disproportionate amount of the variation attributable to hospital-level differences in large community hospitals (and to a lesser extent in teaching hospitals) was experienced by a small percentage of inpatients with the longest LOS”.

2. Repeat Hospitalizations:

***Reviewer Comment:*** *The added sensitivity analysis for repeat hospitalizations is valuable. Expand on how this affects the interpretation of institutional-level variation (e.g., whether certain hospitals disproportionately serve high-frequency users).*

**Author Response:** We have added the following to the discussion on lines 336-339:

“Additionally, the sensitivity analysis looking at repeat hospitalizations found that individuals who had more than 1 hospitalization within the study period had higher proportions of LOS variation attributable to hospital-level differences in large community and teaching hospitals (but not in specialty mental health hospitals).”

## Citation and Referencing

1. Update References:

***Reviewer Comment:*** *Some citations (e.g., Chen et al., 2017) are outdated. Include more recent literature on LOS variation in mental health care.*

**Author Response:** To our knowledge the 2017 paper by Chen et al. is the most directly comparable paper to the work that we are presenting in this paper (being conducted in Ontario and assessing differences in LOS–albeit not variation specifically–between types of hospital). While more recent work related to mental health care related-LOS in different populations (e.g., Valder, Reif, & Tauchmann, 2024, doi: 10.1002/hec.4920; or Chen et al., 2021, doi: 10.1097/MD.0000000000025206), we are not aware of any more recent literature that directly assesses variation in LOS of hospitalizations for schizophrenia.

***Reviewer Comment:*** *Clarify the source of hospital classifications (e.g., Ontario’s Mental Health Act) with specific citations.*

**Author Response:** This question is addressed in our response to the reviewer comment labeled methodology–clarify hospital clarification.

# Reviewer #3

***Reviewer Comment:*** *I congratulate the authors for such a wonderful job and hard work for such informative study. The study has a unique context and concept I understand, yet I would recommend to add 'Acknowledgement' and extract 'Conclusion' after Discussion section at the end. This is recommended for the readability of common people and aspiring future research enthusiasts.*

**Author Response:** We have added the following conclusion to the paper:

“In this study, we analyzed administrative health data to examine the variation in LOS for individuals hospitalized with schizophrenia across different types of hospitals. We found significant variation in LOS among hospitals of all types, highlighting opportunities to optimize healthcare resource use. Addressing this variation could involve organizational and system-level policies, such as standardizing care delivery and enhancing access to community resources like housing. However, further research is needed to identify the key factors contributing to variation in LOS and to evaluate evidence-based care pathways for this patient population.”

***Reviewer Comment:*** *Rest of the comments have been addressed respectively in the first revision. Good luck.*

**Author Response:** Thank you for taking the time to review our paper again, and for your kind words regarding our work.
